# Sequence-Encoded Spatiotemporal Dependence of Viscoelasticity of Protein Condensates Using Computational Microrheology

**DOI:** 10.1021/jacsau.4c00740

**Published:** 2024-11-11

**Authors:** Dinesh Sundaravadivelu Devarajan, Jeetain Mittal

**Affiliations:** 1 Artie McFerrin Department of Chemical Engineering, Texas A&M University, College Station, Texas 77843, United States; 2 Department of Chemistry, Texas A&M University, College Station, Texas 77843, United States; 3 Interdisciplinary Graduate Program in Genetics and Genomics, Texas A&M University, College Station, Texas 77843, United States

**Keywords:** protein condensates, intrinsically disordered proteins, phase separation, microrheology, viscosity, viscoelasticity

## Abstract

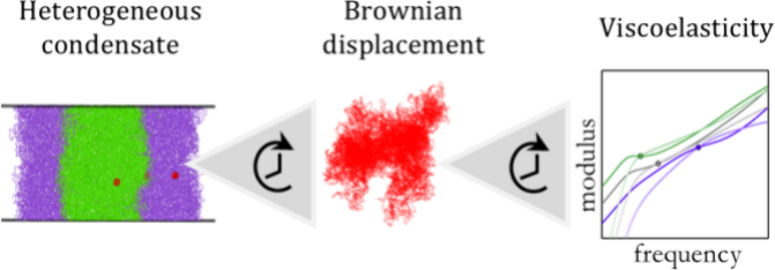

Many biomolecular condensates act as viscoelastic complex fluids with distinct cellular functions. Deciphering the viscoelastic behavior of biomolecular condensates can provide insights into their spatiotemporal organization and physiological roles within cells. Although there is significant interest in defining the role of condensate dynamics and rheology in physiological functions, the quantification of their time-dependent viscoelastic properties is limited and is mostly done through experimental rheological methods. Here, we demonstrate that a computational passive probe microrheology technique, coupled with continuum mechanics, can accurately characterize the linear viscoelasticity of condensates formed by intrinsically disordered proteins (IDPs). Using a transferable coarse-grained protein model, we first provide a physical basis for choosing optimal values that define the attributes of the probe particle, namely, its size and interaction strength with the residues in an IDP chain. We show that the technique captures the sequence-dependent viscoelasticity of heteropolymeric IDPs that differ in either sequence charge patterning or sequence hydrophobicity. We also illustrate the technique’s potential in quantifying the spatial dependence of viscoelasticity in heterogeneous IDP condensates. The computational microrheology technique has important implications for investigating the time-dependent rheology of complex biomolecular architectures, resulting in the sequence–rheology–function relationship for condensates.

## Introduction

Characterizing the spatiotemporal evolution of biomolecular condensates is crucial for understanding their role in modulating cellular biochemistry^1−3^ and how they transform into pathological aggregates.^4−6^ Liquid–liquid phase separation through multivalent interactions in a protein sequence can drive the formation of these condensates.^7−11,^^12^ The liquid-like (viscous) behavior of these cellular compartments is thought to define their functional landscape, by enabling extreme dynamics^13^ and efficient transport of biomolecules that can aid in biochemical processes.^14^ However, recent investigations have demonstrated the loss of condensate liquidity over time, yielding dominant elastic behavior that may eventually promote the formation of solid fibrillar states.^15−17^ Thus, it is critical to accurately quantify the viscoelastic spectrum of biomolecular condensates to establish how the protein sequence governs their (dys)functional paradigm.^18^

Transitions in the material states of protein condensates can occur due to a multitude of factors, e.g., post-translational modifications that alter sequence charge patterning or mutations that alter sequence hydrophobicity.^19−21^ Such sequence alterations commonly occur in intrinsically disordered proteins (IDPs) or regions (IDRs), which are prevalent in condensates, thereby causing a speedup or slowdown in their dynamics. Deciphering the sequence-encoded molecular interactions of IDPs that dictate prominent dynamical changes in conjunction with the measurements of viscoelasticity is essential for establishing the sequence–rheology–function relationship of condensate biology. Simulations can serve as a computational lens into the molecular interactions of condensates and a tool for accurately measuring their rheological properties. For example, the viscosity and viscoelasticity of IDP condensates can be quantified using equilibrium molecular dynamics (MD) simulations along with the Green–Kubo (GK) relation and nonequilibrium MD (NEMD) simulations.^22−26^ While these techniques are useful for measuring the bulk rheology of single-component systems, they suffer from the inability to capture the spatial variations in viscoelasticity seen in multicomponent in vitro and in vivo condensates. Knowledge of the spatial dependence of viscoelastic properties within the condensates can provide important insights for studying how the partitioning and transport of small drug molecules into condensates depends on their local environment.^27^

Particle tracking experimental microrheology is widely used for investigating the time-dependent bulk viscoelasticity and viscosity of in vitro phase-separated droplets displaying different material characteristics ranging from liquid-like to solid-like behaviors.^28−33^ It is a highly sought-after method because it requires only small volumes of biological samples and enables a label-free approach where the biomolecules need not be fluorescently tagged.^34,35^ In addition, the technique has the potential to quantify the local viscoelasticity of heterogeneous condensate systems.^34^ The technique relies on connecting the probe particle motion in a complex fluid system and its microscopic viscoelastic properties through continuum mechanics.^36,37^ A computational analogue of the technique via MD simulations has been shown to yield quantitatively accurate viscoelastic modulus for the homopolymer and colloidal systems,^38−41^ but its rigorous implementation remains untested for the heteropolymeric protein condensates. The success of the computational microrheology technique relies on carefully choosing the parameters for the probe particle, namely, its size and interactions with the medium of interest, such that they follow continuum mechanics assumptions. This is also a primary concern in experimental microrheology where the nonspecific interactions between probe beads and protein molecules need to be prevented for reliable measurements.^35^ Establishing a physical basis for choosing the attributes of the probe is important because of the unexplored questions regarding the technique’s capability in the following two aspects: (1) capturing the sequence-dependent viscoelasticity of condensates formed by heteropolymeric IDPs and (2) quantifying the spatial variations in viscoelasticity found in heterogeneous condensates formed by a pair of heteropolymeric IDPs. To this end, we systematically demonstrate in this work that computational microrheology is a robust technique for studying the time-dependent rheological properties of protein condensates.

## Results

**Figure 1:**
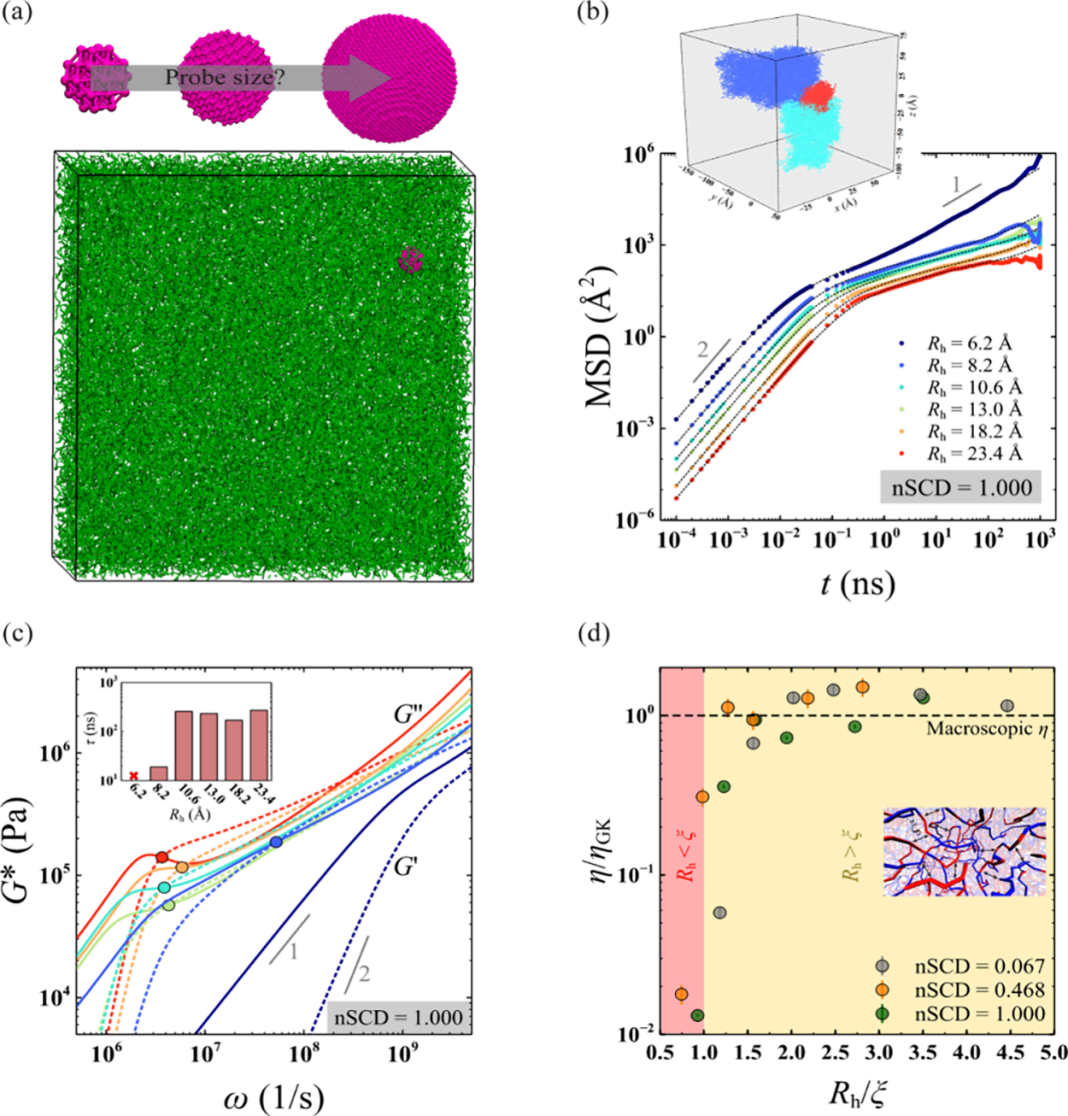
(a) Simulation snapshot of the dense phase of a condensate formed by a select E–K sequence (nSCD = 1) of chain length *N* = 250 with a spherical probe particle (magenta) of hydrodynamic radius *R*_h_ embedded in it. (b) Mean square displacement MSD(*t*) of the center of mass of the probe particle for different *R*_h_ values in the dense phase of the E–K sequence with nSCD = 1. The dashed lines are the fits based on the Baumgaertel–Schausberger–Winter-like power law spectrum to the MSD data. The inset shows the probe’s displacement in the three-dimensional Cartesian coordinates for select *R*_h_ values. (c) Elastic *G*^′^ (dashed line) and viscous *G*^″^ (solid line) moduli for the dense phase of the E–K sequence with nSCD = 1 as a function of *R*_h_, with circles corresponding to the crossover frequency. The inset shows the relaxation time τ, computed as the inverse of the crossover frequency, with varying *R*_h_. (d) Viscosity η, normalized by that obtained based on the Green–Kubo (GK) relation η_GK_, as a function of normalized probe particle size *R*_h_/ξ, where ξ is the correlation length, for three different nSCD sequences. The two shaded regions delineate regions where *R*_h_ is smaller than or larger than ξ. The black dashed line corresponds to η = η_GK_.

To investigate the sequence-dependent viscoelasticity of IDP condensates using computational microrheology, we employed coarse-grained (CG) model sequences formed by an equal number of negatively charged glutamic acid (E) and positively charged lysine (K) with chain lengths of *N* = 250 and *N* = 50 residues. Recently, we used similar IDP sequences to establish the sequence-dependent material properties, namely, diffusion coefficient *D* and viscosity η of condensates formed by a charge-rich model and naturally occurring IDPs.^24^ We chose three E–K sequences that varied in the sequence charge patterning, with a high degree of charge segregation quantified using a high value of normalized sequence charge decoration (nSCD ∈ [0,1]) parameter^42−44^ (see Table S1 for the amino acid sequences). Specifically, we used variants with nSCD values of 0.067, 0.468, and 1.000, respectively. We performed computational passive probe microrheology via MD simulations in a cubic simulation box at the preferred dense phase concentrations of the E–K variants (see Methods). We found that the dense phase concentration ρ increased with increasing degree of charge segregation (i.e., increasing nSCD), highlighting stronger effective interactions between the oppositely charged residues (Figure S1). A probe particle of bare mass *m*_bare_, modeled as a rough sphere for ensuring no-slip boundary conditions,^45^ was dispersed in the dense phase of the E–K variants (Figure 1a). All the simulations were carried out via a transferable hydropathy scale (HPS) model^46,47^ at a constant temperature of *T* = 300 K (see Methods for model and simulation details).

The friction encountered by the probe particle during its Brownian motion can be related to the linear viscoelastic properties (elastic *G*^′^ and viscous *G*^″^ modulus) of the dense phase of a condensate using the inertial generalized Stokes–Einstein relation (IGSER).^48,49^
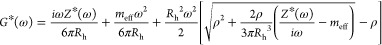
1where G* = *G*^′^ + *iG*^″^ is the dynamic modulus of the medium, ω is the frequency, Z* is the frequency-dependent friction experienced by the probe through its interactions with the medium, *R*_h_ is the hydrodynamic radius of the probe, and *m*_eff_ is the effective mass of the probe particle. As per continuum mechanics, *m*_eff_ = *m*_bare_ + *m*_add_, where  is the added mass from the medium.^50^ For IGSER to accurately capture the viscoelastic properties of IDP condensates, two important characteristics of the probe emerge, namely, its size (i.e., *R*_h_) relative to the relevant length scale of the IDP dense phase and its interactions with the IDP chains. This prompted us to do a systematic assessment of the attributes of the probe particle for the successful implementation of computational microrheology for protein condensates.

### Probe Particle Size is a Critical Determinant for the Computational Microrheology of IDP Condensates

Given that the length of our model E–K sequences is representative of naturally occurring IDPs, we first asked whether there is an appropriate size of the probe particle, which can be rationalized in terms of the relevant length scale such as the mesh size within IDP condensates.^51^ To investigate this aspect, we varied the probe sizes, ranging from a bare radius of *R*_b_ = 2.5 Å (similar to the radius of the residue beads in the HPS model) to *R*_b_ = 20 Å (comparable to the dense phase radius of gyration *R*_g_ of the E–K variants with *N* = 50).^44^ For our continuum mechanics analysis, we used the hydrodynamic radius *R*_h_ of the probe particle, defined as the location of the first peak in the radial distribution function (RDF) between the probe and the protein residues within the dense phase of the condensates (Figure S2). This choice of definition for *R*_h_ was previously shown to recover the Stokes frictional force and torque for the rough spherical particle moving through a complex fluid as well as yield accurate *G*^′^ and *G*^″^ values of homopolymers with increasing *N*.^38,39,45^ For all sizes of the probe, its interaction with the protein residues was modeled via a modified Lennard-Jones potential with interaction strength ε_HPS_ = 0.2 kcal/mol, the strongest possible van der Waals interaction between a pair of residue beads in our HPS model.

In our passive rheology simulations, we tracked the Brownian displacement of the center of mass of the probe of different sizes in the dense phase of the E–K sequences (insets of Figure 1b and Figures S3a and S4a). Using the displacement data, we then computed the probe’s mean square displacement
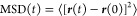
2where ***r*** is its position at time *t* (Figure 1b and Figures S3a and S4a). We found that probes of all sizes *R*_h_ exhibited a ballistic motion (MSD ∝ *t*^2^) at short times, but only the smallest probe (*R*_h_ = 6.2 Å) showed a diffusive motion (MSD ∝ *t*) at long times. We observed a subdiffusive behavior at intermediate times, which became increasingly prominent until long times with increasing *R*_h_, indicating that larger probes move slower in the condensate within the simulation duration of the E–K sequences investigated. Furthermore, MSD at intermediate to long times drastically decreased for smaller *R*_h_, followed by a gradual decrease for larger *R*_h_, highlighting the high friction exerted on large probe particles. In fact, the friction Z* that appears in IGSER can be related to the MSD of the probe as
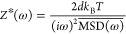
3where *d* is the number of dimensions in which the particle is tracked, *k*_B_ is the Boltzmann constant, and MSD(ω) is the one-sided Fourier-transformed MSD. For obtaining MSD in the frequency domain, we used a previously developed analytical fitting procedure by expressing MSD in terms of a Baumgaertel–Schausberger–Winter-like power law spectrum (see Methods; Figure 1b and Figures S3a and S4a).^39,52^ This method is usually superior to the use of an approximate Fourier transform expression used for the polynomial fit to MSD(*t*) in experimental microrheology,^37^ which often leads to poor estimates when the slope of MSD varies rapidly.

Next, we used IGSER (eqs 1 and 3) to obtain the elastic *G*^′^ and viscous *G*^″^ moduli of the dense phase of E–K sequences (Figure 1c and Figures S3b and S4b). Consistent with the trends in the probe motion, we found that *G*^′^ and *G*^″^ were significantly lower and that the terminal regime (i.e., *G*^′^ ∝ ω^2^ and *G*^″^ ∝ ω^1^) was observed at high frequencies for smaller probe particles (i.e., *R*_h_ ≤ 8.2 Å) as compared to the larger ones for all E–K sequences. For *R*_h_ > 8.2 Å, the onset of terminal viscous characteristics occurred at similar frequency values, as indicated by similar crossover frequencies below which *G*^″^ > *G*^′^. This was also evident by further looking at the similar relaxation times τ, computed as the inverse of crossover frequency, for larger probe particles in the dense phase of E–K variants (insets of Figure 1c and Figures S3b and S4b). Our findings indicate that for probe sizes beyond a certain threshold value (i.e., *R*_h_ > 8.2 Å), the probe motion yields a viscoelastic spectrum of the dense phase of E–K sequences that exhibit similar characteristics (i.e., similar τ).

Given that the motion of the probe is closely tied to the dense environment of the IDP condensates, we next investigated whether the probe sizes (*R*_h_ > 8.2 Å) that yielded similar viscoelastic spectrum were larger than the mesh size^53^ within the dense phase of E–K sequences. Note that the IGSER requires the probe particle to be large enough to see the medium as a continuum. Following a previous work,^51^ we estimated the correlation length ξ from the overlap concentration ρ*, which is related to the polymer mesh size in a dense phase system (see Methods). We found the values of ξ to be in the range of 5.24 Å to 8.32 Å for the E–K sequences. We also computed the viscosity of the dense phase of E–K sequences from the microrheology simulations as η = *G*^″^/ω in the terminal region as well as from the GK relation that yields macroscopic viscosity η_GK_ (see Methods). When we normalized η by η_GK_, we found that probes larger than the correlation length (i.e., *R*_h_/ξ ≳ 1.5) yielded macroscopic viscosity (Figure 1d). This finding highlights that probes larger than ξ feel the “true” macroscopic friction present within the dense phase. However, given that very large probes sample the dense phase of the condensates much more slowly (insets of Figure 1b and Figures S3a and S4a), we concluded that the smallest probe size that satisfied the criteria *R*_h_/ξ ≈ 1.5 (i.e., *R*_h_ = 10.6 Å) would be a computationally efficient choice to obtain reliable viscoelastic measurements of any IDP condensates investigated using the HPS model and other analogous CG models.^54−57^

### Confluence of Probe Particle Size and Its Interaction Strength with IDPs Correctly Captures Condensate Rheology

**Figure 2:**
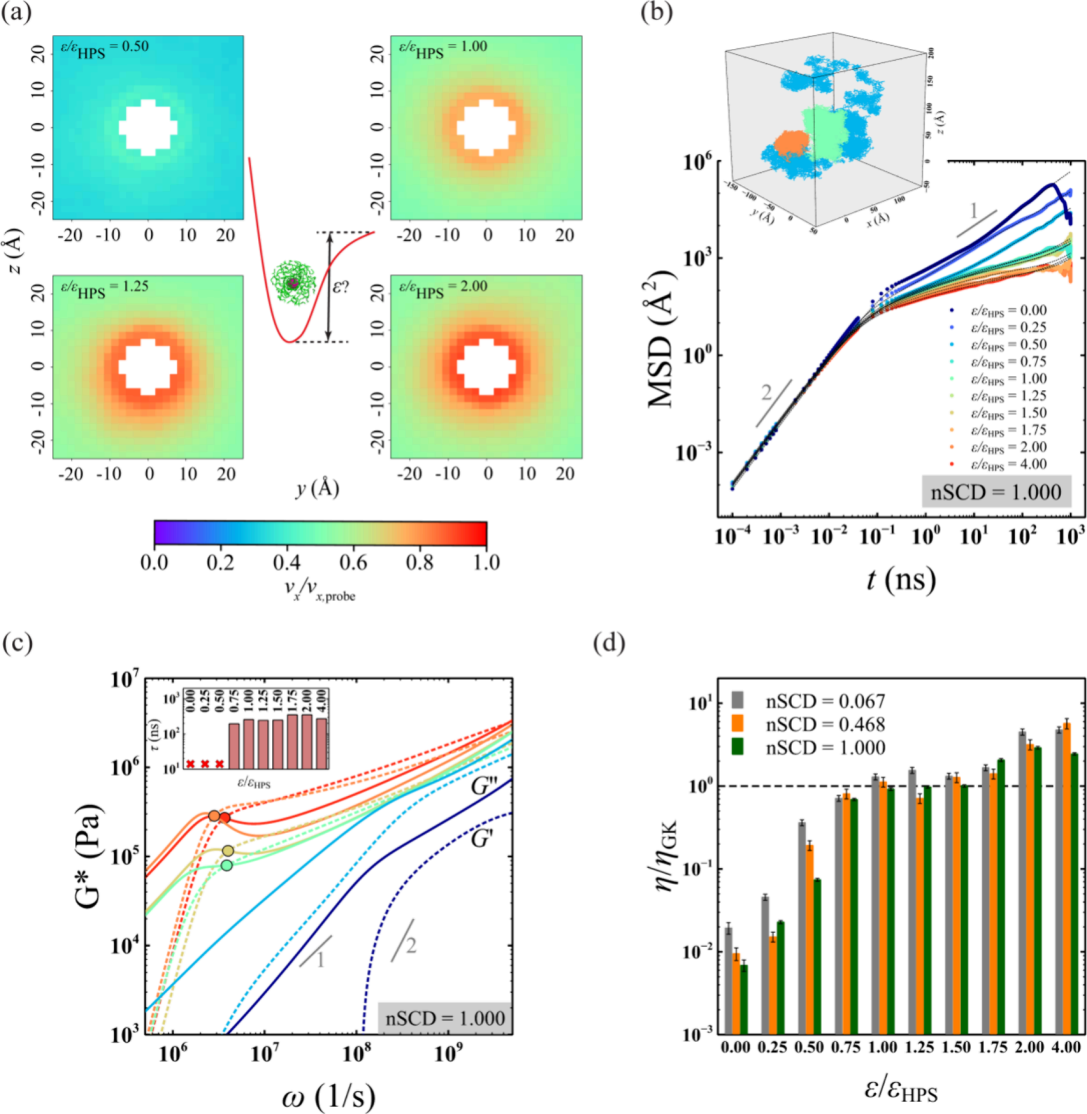
(a) Velocity *v*_*x*_ profile of the residues of protein chains with nSCD = 1 around the probe particle translating with a velocity *v*_*x*,probe_ for different probe–protein interaction strengths ε, normalized by the strongest possible van der Waals interaction strength ε = 0.20 kcal/mol in the HPS model. (b) Mean square displacement MSD(*t*) of the center of mass of the probe particle for different ε/ε_HPS_ in the dense phase of the E–K sequence with nSCD = 1. The dashed lines are the fits based on the Baumgaertel–Schausberger–Winter-like power law spectrum to the MSD data. The inset shows probe’s displacement in the three-dimensional Cartesian coordinates for select ε/ε_HPS_ values. (c) Elastic *G*^′^ (dashed line) and viscous *G*^″^ (solid line) modulus for the dense phase of the E–K sequence with nSCD = 1 as a function of ε/ε_HPS_, with circles corresponding to the crossover frequency. The inset shows the relaxation time τ with varying ε/ε_HPS_. (d) Viscosity η, normalized by that obtained based on the Green–Kubo (GK) relation η_GK_ as a function of ε/ε_HPS_ for three different nSCD sequences. The black dashed line corresponds to η = η_GK_.

The friction experienced by the probe particle is dependent on its interaction with the IDP chains constituting the condensates. Having established a suitable probe size for computational microrheology, we next investigated whether the strength of the probe–protein interactions had a significant effect on the viscoelastic modulus of the dense phase of IDP condensates. An important requirement imposed by IGSER is the need for a no-slip boundary condition at the probe particle surface.^39,40^ To identify the interaction strengths ε that would satisfy the condition, we varied it in the range of ε/ε_HPS_ = 0 (purely repulsive interactions) to ε/ε_HPS_ = 4 for a probe particle size of *R*_h_ = 10.6 Å. We then computed the velocity *v*_*x*_ profile of the protein residues around the probe particle that was moving at a predefined translational velocity *v*_*x*,probe_ (Figure 2). We found that the velocity of the protein residues adjacent to the probe surface became increasingly similar to that of *v*_*x*,probe_ with increasing ε/ε_HPS_, indicating that sufficient attractive interactions prevent a slip at its surface. In line with this observation, we observed that only probe particles with ε/ε_HPS_ < 0.75 exhibited very high displacements as well as a diffusive behavior within the course of the simulations of E–K sequences (Figure 2b and Figures S5a and S6a). To quantify the variations in the probe’s MSD for different ε/ε_HPS_ in terms of viscoelasticity, we measured *G*^′^ and *G*^″^ of the dense phase of E–K variants via IGSER (Figure 2c and Figures S5b and S6b).

For probes with ε/ε_HPS_ < 0.75, we found that *G*^′^ and *G*^″^ were significantly lower and did not show a crossover between them as compared to the higher ε/ε_HPS_ values. When sufficient attractive interactions (ε/ε_HPS_ ≥ 0.75) exist between the probe and the protein residues, similar relaxation time scales delineating dominant viscous and elastic behaviors were observed irrespective of ε/ε_HPS_ for all sequences (Figure 2c and Figures S5b and S6b). However, for ε/ε_HPS_ = 4, the modulus values were much higher compared to ε/ε_HPS_ = 1. This is further highlighted in the normalized viscosity η/η_GK_ values, which revealed an optimal window for interaction strengths (1 ≤ ε/ε_HPS_ ≤ 1.5) that yielded macroscopic viscosity (Figures 2d). Note that, for ε/ε_HPS_ ≳ 1.75, we observed the IDP chains getting strongly adsorbed to the probe particle surface (Figure S7). This is a common concern in experimental microrheology as well, in which, for example, polystyrene beads are often passivated with polyethylene glycol, to ensure negligible chemical interactions with the biomolecules as well as to prevent the beads from getting constrained within the droplet.^35,58,59^ From these findings, we concluded that an attractive strength (ε/ε_HPS_ = 1) that is low enough but a value that resides in the optimal window would be ideal for correctly capturing the time-dependent rheology of IDP condensates computationally.

### Computational Microrheology Reveals the Sequence-Encoded Time-Dependent Viscoelasticity of Heteropolymeric IDP Condensates

**Figure 3:**
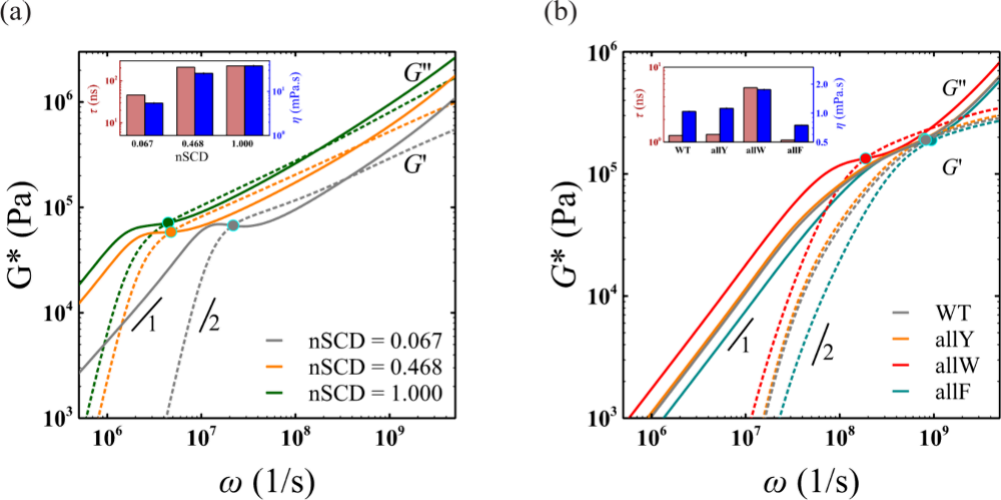
(a) Elastic *G*^′^ (dashed line) and viscous *G*^″^ (solid line) modulus for three E–K sequences with different nSCD, obtained based on multiple (*n* = 8) probes in each dense phase system. (b) Elastic *G*^′^ (dashed line) and viscous *G*^″^ (solid line) modulus for A1-LCD wild-type (WT) and its three variants, obtained based on multiple (*n* = 6) probes in each dense phase system. The circles in (a) and (b) correspond to the sequence-specific crossover frequencies. The insets in (a) and (b) show the changes in relaxation time τ and viscosity η with changing nSCD and with mutational changes in the A1-LCD WT sequence, respectively.

Through the continuum analysis of the motion of a single probe particle in our computational microrheology simulations, we have identified a suitable probe particle size and its interaction strength with the IDP chains that would yield accurate macroscopic dense phase viscosities of the E–K sequences. However, the microrheology experiments are often performed with multiple probe particle beads within the same droplet, and the average motion of all particles is used for quantifying the condensate viscoelasticity.^13,28^ This approach is efficient, as it reduces the uncertainties in the viscoelastic measurements, which may arise from tracking only a single particle. Although the continuum mechanics expressions are for a single particle, which necessitates that no hydrodynamic interactions exist between the probe particles, we next asked the extent to which multiple probes in an IDP dense phase system would influence their motion, which can alter the corresponding viscoelastic measurements. For this purpose, we performed the passive rheology simulation with *n* = 2 to 48 probes in the dense phase of E–K sequences (Figure S8). Surprisingly, we found that the probe’s average MSD was nearly the same at all times with varying *n*, but the statistical noise in the MSD profile has significantly reduced for systems with multiple particles. To demonstrate the ability of passive rheology simulations to capture the changes in viscoelasticity with increasing nSCD, we again obtained the viscoelastic moduli *G*^′^ and *G*^″^ of the dense phase of E–K sequences based on the average MSD of *n* = 8 probes (Figure 3a). The modulus values obtained from the microrheology simulations using multiple probes are in quantitative agreement with those obtained based on a single probe particle (Figure S9). Furthermore, we also observed a semiquantitative agreement, yet similar trends in the modulus values obtained from the microrheology simulations and based on the Green–Kubo relation for all E–K sequences, even though the latter method suffers from noise at long times (see Methods; Figures S10 and S11). Our microrheology simulations revealed that the modulus *G*^′^ and *G*^″^, relaxation time τ, and viscosity η were higher with increasing nSCD, highlighting that charge segregation slows down dynamics, thereby resulting in longer time scales for displaying the terminal flow behavior (Figure 3a). Finally, we also tracked the probe motion in the dense phase of E–K sequences of *N* = 50,^24^ but with the same nSCD values as those used for *N* = 250 (Figure S12a). We found that *G*^′^ and *G*^″^ did not show a crossover (i.e., no dominant elastic response in the entire frequency space) for the shorter sequences, indicating negligible entanglement effects between the chains in these systems (Figure S12b). Our findings indicate that condensates formed by longer chains exhibit Maxwell fluid-like behavior over the entire frequency range investigated, which is in agreement with recent experimental^28^ and computational studies.^60,61^

The dynamics and rheology of IDP condensates can be modulated not only through sequence charge patterning but also via alterations to the sequence hydrophobicity. We next investigated the ability of the passive rheology technique coupled with our CG model to capture sequence alterations affecting the hydrophobic character. To this end, we tracked multiple (*n* = 6) probe particles in a naturally occurring IDP A1-LCD wild-type (WT) sequence and three of its variants, each with *N* = 137, with different aromatic residue (tyrosine Y, tryptophan W, and phenylalanine F) identities, namely, allY, allW, and allF (see Table S2 for the amino acid sequences). Again, we ensured that the average probe MSD computed based on *n* = 6 particles was similar to the MSD of a single particle in these systems, except for long times where the statistics for the MSD based on multiple probes were better as compared to a single-probe MSD (Figure S13). The values of *G*^′^ and *G*^″^, τ, and η obtained using IGSER increased in the following order: allW > allY ≈ WT > allF (Figure 3b). This finding is in agreement with the recent experimental microrheology measurements on the same set of IDP sequences.^62^ We did not attempt to make a quantitative comparison of the rheological properties obtained from CG simulations and experiments due to the orders of magnitude discrepancy between them (see Table S3), which is expected and arises from the use of the intrinsic MD time scale.^24,63^ It is important to note that this quantitative discrepancy is not due to the computational microrheology method itself. In fact, we expect that applying the microrheology technique to all-atom models would yield quantitatively accurate rheological properties. In conclusion, we believe that the computational microrheology technique, when coupled with continuum mechanics and CG models, can accurately predict sequence-encoded, time-dependent viscoelastic changes in IDP condensates.

### Computational Microrheology Unmasks the Spatial Variations in the Viscoelasticity of Heterogeneous IDP Condensates

**Figure 4:**
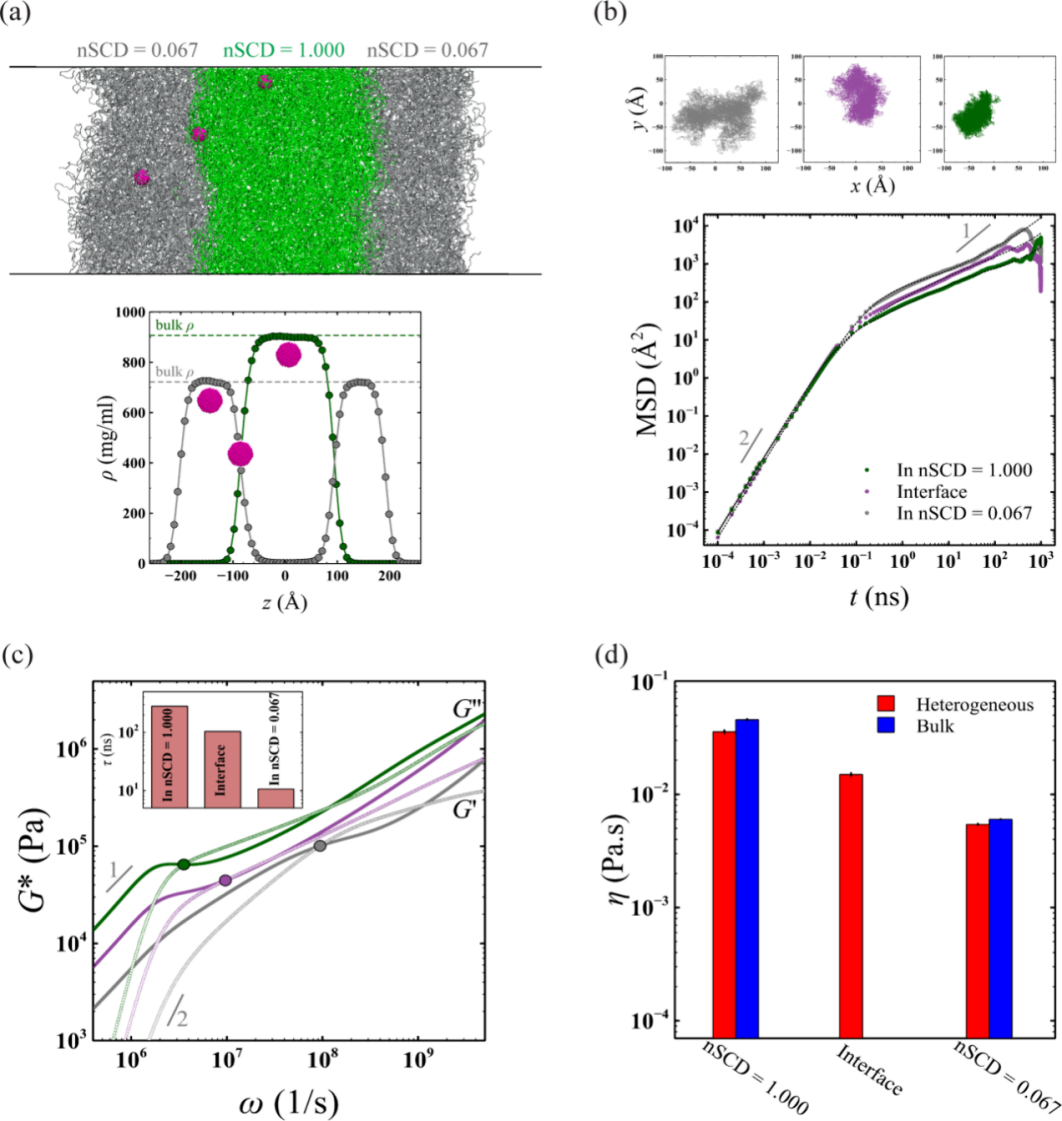
(a) Simulation snapshot of a heterogeneous condensate formed by two different E–K sequences with nSCD = 0.067 and nSCD = 1.000. Three probe particles are spatially restrained in three regions of the heterogeneous condensate: one among nSCD = 1.000 chains, one among nSCD = 0.067 chains, and one at the interface of the two nSCD sequences. Also shown are the density profiles of the heterogeneous condensate. (b) Mean square displacement MSD(*t*) of the probe particle in three different regions of the heterogeneous condensate. The dashed lines are the fits based on the Baumgaertel–Schausberger–Winter-like power law spectrum to the MSD data. The inset shows the probe’s displacement in the two-dimensional Cartesian coordinates in the chosen regions within the condensate. (c) Elastic *G*^′^ (open symbols) and viscous *G*^″^ (closed symbols) modulus sampled from three different regions of the heterogeneous condensate, with circles corresponding to the location-specific crossover frequency. The inset shows the relaxation time τ in the chosen regions within the condensate. (d) Viscosity η in three regions of the heterogeneous condensate compared to those obtained from the bulk simulations of the E–K sequences.

Intracellular biomolecular condensates are multicomponent in nature, often exhibiting complex molecular architectures with spatial heterogeneities.^64^ We next asked whether computational microrheology can be used to quantify the spatial variations in the viscoelasticity of heterogeneous condensates. It is known that IDPs with large differences in nSCD values demix in such a way that a highly charge-segregated sequence forms the condensate core and the well-mixed sequence forms a shell around the core.^65^ Specifically, we formed such a condensate using a pair of E–K sequences with nSCD = 0.067 and 1.000 (Figure 4). Also, the maximum densities of the two E–K sequences in the heterogeneous mixture were the same as those observed in the bulk condensates simulated in a cubic geometry (Figure 4a). To characterize the local viscoelasticity within this heterogeneous condensate, we spatially restrained *n* = 3 probes along the *z* direction encompassing distinct local environments: one among nSCD = 1.000 chains, one among nSCD = 0.067 chains, and one at the interface of the two nSCD sequences (see Methods). We found that the two-dimensional probe particle displacements were different in each of the three regions at intermediate to long times, with the probes among nSCD = 0.067 chains and nSCD = 1.000 chains exhibiting the highest and lowest mobilities, respectively (Figure 4b). This was evident in the elastic *G*^′^ and viscous *G*^″^ modulus obtained by using the two-dimensional probe particle displacement information in IGSER (Figure 4c). Specifically, the values of modulus and the corresponding relaxation times τ were lower for the shell region around the condensate core than for the core itself, while the values at the interface between the core and the shell regions displayed intermediate viscoelasticity. Interestingly, we also found that viscosity η values at the condensate core and shell regions, obtained based on *G*^″^ in the terminal region, were quantitatively similar to that obtained from the bulk condensates (Figure 4d). These observations further highlight the potential of computational microrheology for revealing the location-dependent viscoelasticity of complex heterogeneous condensates, which can provide insights about the small molecules that partition into such environments^27^ as well as help define the role of condensate interfaces that have been found to exhibit distinct conformational characteristics as compared to the core of the condensates.^66,67^

## Conclusions

Knowledge of the molecular interactions that govern the viscoelastic transitions would allow for establishing how the protein sequence dictates the spatiotemporal evolution of condensates. Using the HPS model, we demonstrate that a computational passive probe microrheology technique, in which the probe particle motion is analyzed via continuum mechanics, can accurately quantify the sequence-dependent viscoelasticity of IDP condensates. We do so by first rationalizing our choice for the two important attributes of the probe particle, namely, its size and interaction strength with the IDPs. We found that a probe with a hydrodynamic radius *R*_h_ being slightly greater than the correlation length ξ within the condensates (*R*_h_ ≈ 1.5ξ) and its interactions with the IDPs being optimally strong that it prevents slip (ε/ε_HPS_ = 1) is sufficient to accurately quantify the viscosity and viscoelasticity of IDP condensates. Given that the probe size and its interaction strength with the protein residues are interdependent, selecting too small a probe size with strong attractive interactions or too large a probe size with strong repulsive interactions could disrupt the inherent mesostructure of the condensates. Therefore, we believe that the values we have selected are optimal for accurately characterizing the rheology of protein condensates. Experimental microrheology employs probe particles that are significantly larger than those used in our simulations.^32^ The IGSER relation also assumes that the probe particle is large enough to treat the medium as a continuum. However, previous studies on experimental microrheology of entangled DNA solutions^68^ and computational microrheology of weakly entangled polymer melts^39^ have shown that probe sizes between 1.5*d*_T_ – 3*d*_T_ (*d*_T_ is the entanglement tube diameter) can still yield accurate measurements of viscoelastic properties. Using very large probe particles in simulations, however, would risk trapping the probe within the condensate, preventing it from sufficiently sampling the local environment. Thus, for other CG models with similar resolution, we expect that estimating ξ and selecting a probe size between 1.5ξ and 2ξ should yield accurate results. Similarly, a good starting point for the probe’s interaction strength with the protein chains is to use the strongest possible vdW interaction and compute velocity profiles around the probe by translating it at a fixed velocity within the condensate to verify that the no-slip boundary condition holds. By tracking the motion of the probe particle and converting its displacement information into viscoelastic modulus using IGSER, we found that the measurements from microrheology simulations are in quantitative agreement with those obtained based on the Green–Kubo (GK) relation. Furthermore, the elastic and viscous modulus, relaxation time, and terminal viscosity increased with increasing degree of charge segregation, exhibiting a Maxwell fluidic nature for the E–K sequences with identical sequence compositions. This observation highlighted that computational microrheology captures the time-dependent rheological transitions with sequence alterations that result in pronounced electrostatic interactions between the oppositely charged residues. Furthermore, we have shown that the microrheology simulations can capture changes in viscoelasticity with changes in the composition of aromatic residues in the naturally occurring A1-LCD WT sequence through mutations. Taken together, we conclude that computational microrheology is a robust technique for characterizing the sequence-encoded time-dependent viscoelasticity of heteropolymeric protein condensates.

Microrheology experiments are often performed with multiple probe particles within the same droplet, and the average displacement of the particle is used to characterize condensate viscoelasticity. We also showed that microrheology simulations performed with multiple probe particles yield accurate viscoelastic modulus values of the dense phase of the IDP sequences investigated in this work. This suggests that a single simulation with multiple probes can lead to improved statistical accuracy of the probe’s displacement at long times. We note that the largest number of probes (*n* = 48) used in this work corresponds to a volume fraction of 0.1. This value is approximately the maximum volume fraction at which Einstein’s viscosity equation remains valid for nanoparticle suspensions;^26^ beyond this point, nanoparticle–nanoparticle interactions dominate, leading to an exponential increase in viscosity. Therefore, we expect that a probe volume fraction below 0.1 would yield reliable viscoelastic measurements of condensates from passive rheology simulations regardless of the CG models used. However, we recommend using a lower-volume fraction, similar to the ones used for obtaining the viscoelastic properties of E–K and A1-LCD sequences in Figure 3. Consequently, condensate viscoelasticity can be sampled based on a single simulation trajectory as opposed to other conventional techniques such as the MD simulations along with the GK relation and the NEMD simulations. In the method involving GK relation, the shear stress relaxation modulus used to obtain the viscoelastic modulus typically requires extensive statistics, which can only be achieved through long simulations to accurately capture its decay over time.^22^ In the NEMD method, viscoelastic modulus can only be obtained by applying an oscillatory shear strain on the system at a specific frequency and is prohibitive of accessing low-frequency (long-time) viscoelasticity, rendering it computationally expensive.^26,38^ Furthermore, the use of multiple probe particles can allow for the accurate characterization of viscoelasticity in biomolecular condensates with inherent heterogeneities due to physical aging.^62,69^ Finally, we spatially restrained multiple-probe particles at distinct locations within a heterogeneous IDP condensate formed by a pair of E–K sequences with vastly different charge patterns for quantifying local viscoelasticity. By tracking each of the particle’s displacements in unrestrained directions, we demonstrated the technique’s ability to accurately quantify the viscoelasticity that depends on the local environment within such condensates. This finding highlights that passive rheology can be used for accurately sampling the spatial variations in viscoelasticity that are prevalent in intracellular condensates. Knowledge of the spatial dependence of viscoelasticity can provide important insights for designing drug-delivery nanoparticles with targeted partitioning and transport within condensates.^70^ We believe that the computational microrheology technique can be an easy-to-use technique for establishing the sequence determinants of condensate viscoelasticity for a wide range of protein condensates.

## Methods

### Hydropathy Scale (HPS) Model

We used our transferable CG model based on the hydropathy scale to computationally investigate the IDP sequences.^46,47^ This CG framework has been widely used in deciphering the sequence-dependent conformations and phase separation of a wide range of IDPs.^44,54,71−76^ In this framework, we represent the IDPs as fully flexible polymeric chains with a single bead per residue representation. Interactions between bonded residues occurred via the harmonic potential
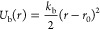
4with distance *r* between residues, spring constant, and equilibrium bond length set to *k*_b_ = 20 kcal/(mol Å^2^) and *r*_0_ = 3.8 Å, respectively. The van der Waals interactions between nonbonded residues *i* and *j* were modeled using the modified Lennard-Jones (LJ) potential^77,78^ based on the average hydropathy λ = (λ_*i*_ + λ_*j*_)/2


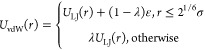
5where *U*_LJ_ is the standard LJ potential
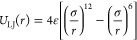
6

The parameters of the LJ potential are the average diameter σ = (σ_*i*_ + σ_*j*_)/2, and the interaction strength ε = ε_HPS_ = 0.2 kcal/mol. We used λ values based on the Kapcha–Rossky (KR) hydropathy scale^79^ for the E–K sequence variants and those based on the Urry hydropathy scale^80^ for the A1-LCD sequence variants. We used the Urry scale for A1-LCD, as it is known to capture the changes in the phase behavior of natural proteins upon mutations of arginine to lysine and tyrosine to phenylalanine.^47^ We used the KR scale for the E–K sequences to maintain consistency with our previous works on these model sequences^24,43,44^ and because the behavior of these sequences is predominantly driven by electrostatic interactions, which are equivalent in both scales. Regardless of the hydropathy scale used for simulating the E–K sequences, the total pairwise energy profiles remain very similar once the electrostatic contributions are accounted for (Figure S14). The values of *U*_vdW_ and its forces were truncated to zero at a distance of 4 σ. Finally, the nonbonded charged residues interacted through a Coulombic potential with Debye–Hückel electrostatic screening^81^
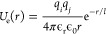
7with vacuum permittivity ϵ_0_, relative permittivity ϵ_r_ = 80, and Debye screening length *l* = 10 Å. The choices for ϵ_r_ and  were made to represent an aqueous solution with a physiological salt concentration of ∼100 mM. The values of *U*_e_ and its forces were truncated to zero at a distance of 35 Å.

### Model of the Probe Particle and Its Interactions with IDPs

We modeled the probe particle by carving out a spherical region from a face-centered cubic (FCC) crystal lattice structure of the LJ beads (σ = 1.5 Å and mass *m* = 100 g/mol), with a lattice spacing (i.e., distance between corner atoms) of 2.12 Å. This value was chosen because it ensures that the corner and face atoms in the FCC lattice are at a distance of 1.5 Å (i.e., just touching each other). The spherical shape of the probe particle was maintained by connecting the neighboring LJ particles, constituting the corner and the face atoms, using stiff harmonic bonds with a spring constant *k*_b_ = 250 kcal/(mol Å^2^). We ensured that *R*_g_ of the probe particle was nearly identical to the expected value for a spherical particle  as well as its relative shape anisotropy κ^2^ was nearly zero (κ^2^ = 0 for a sphere) during the simulations (Figure S15).^53^

The probe particle beads interacted with all protein residues in an IDP chain via the modified LJ potential *U*_vdW_ (eqs 5 and 6), in which λ was varied between the values of 0 and 4 to control the interaction strength ε. Specifically, the values of ε were in the range of 0 to 0.8 kcal/mol. Again, we truncated *U*_vdW_ and its forces to zero at a distance of 4 σ. We note that the interactions between the beads constituting the probe particle were modeled using a purely repulsive potential, which corresponds to λ = 0 in eq 5.

### Microrheology Simulation Details

We simulated the IDP sequences (400 chains of E–K sequences and 800 chains of A1-LCD sequences) in a cubic simulation box at a constant pressure of *P* = 0 atm for a duration of 0.2 μs. At the end of this equilibration run, the IDPs reached their preferred sequence-dependent dense phase concentration ρ. We then performed Langevin dynamics (LD) simulations in the canonical ensemble for a total duration of 1 μs. For these simulations, a damping factor of *t*_damp_ = 1 ns was used to set the friction coefficient of a residue in the chain as well as a bead constituting the probe particle to *f* = *m*/*t*_damp_. Following a previous work,^51^ we computed the correlation length within the dense phase of the condensates as
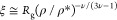
8where ρ* and ν correspond to the overlap concentration and Flory’s scaling exponent, respectively. We estimated the overlap concentration as  with *R*_e_ being the end-to-end distance of protein chains, *M*_w_ being the protein’s molecular weight, and *N*_A_ is the Avogadro constant. We used ν = 1/2 as we found in our previous work that the IDPs closely followed the ideal chain statistics within the dense phase.^44^

For characterizing the spatial dependence of viscoelasticity, we performed LD simulations of a heterogeneous condensate formed by a pair of E–K sequences in a slab geometry (225 Å × 225 Å × 1687.5 Å) for a duration of 1 μs. The friction coefficients for the IDP residues and the probe particle beads were the same as those used in the bulk dense phase simulations. Three probe particles were restrained via a harmonic potential with *k*_b_ = 20 kcal/(mol Å^2^) at specific locations along the *z* direction of the heterogeneous condensate via the restrain functionality (i.e., restrain.plane) within azplugins.^82^

For comparison with the microrheology simulations, we performed equilibrium MD simulations of the E–K sequence variants in the absence of a probe particle to compute their viscosity and viscoelasticity using the Green–Kubo relation
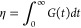
9where *G*(*t*) is the shear stress relaxation modulus. We measured *G*(*t*) (Figure S16) based on the autocorrelation of the pressure tensor components *P*_*ab*_(22,83)
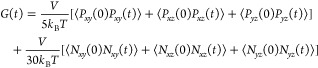
10where *V* is the volume of the simulation box and *N*_*ab*_ = *P*_*aa*_ – *P*_*bb*_ is the normal stress difference. For computing viscosity η, we followed the approach of Tejedor et al.^22^ by fitting the smooth *G*(*t*) profile at long times to a series of Maxwell modes (*G*_*i*_ exp (−*t*/τ_*i*_) with *i* = 1····4) equidistant in logarithmic time.^53^ We obtained η by summing up the values from numerical integration at short times and analytical integration based on the fits to Maxwell modes at long times. For computing elastic *G*^′^ and viscous *G*^″^ modulus, we Fourier-transformed *G*(*t*) using the RepTate software.^84^

All of the simulations were performed with periodic boundary conditions applied to all three Cartesian directions. The simulations were performed with a time step of 10 fs using HOOMD-blue (version 2.9.3)^85^ with features extended using azplugins (version 0.10.1).^82^

### Continuum Analysis of the Probe Motion

In microrheology, the viscoelastic modulus (elastic *G*^′^ and viscous *G*^″^ modulus) is estimated from the probe particle’s motion, which is considered to obey the generalized Langevin equation (GLE)


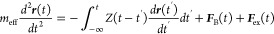
11where *Z*(*t*) is the time-dependent friction, ***F***_B_ is the Brownian force on the probe particle, and ***F***_ex_ is the external force on the probe particle, which is zero in the case of our passive rheology simulations. Because of the low time scales inherent in the MD simulations, inertia plays an important role in accurately quantifying the modulus values of protein condensates. When the inertial terms are included, the GLE in the frequency domain takes the form^38,39,48^
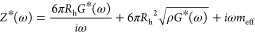
12where the terms on the right side correspond to the generalized Stokes drag, the Basset force arising from the medium inertia, and the effective probe particle inertial force, respectively. In experimental microrheology, the generalized Stokes drag alone is sufficient to obtain the viscoelastic modulus of complex fluid systems. On rearranging eq 12, we obtained IGSER as given in eq 1.

### Analytical Expression Describing the Probe’s Displacement Data

We followed the approach originally proposed by Karim et al.,^38,39^ which we discuss here for the sake of completeness. The probe’s mean square displacement can be described through the Baumgaertel–Schausberger–Winter-like power law spectrum
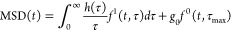
13with *f*^1^, *f*^0^, and τ being the first function for capturing the short-time ballistic behavior (MSD = *Ct*^2^, where *C* is the ballistic coefficient), the second function for capturing the long-time diffusive behavior (MSD = 6*Dt*, where *D* is the diffusion coefficient), and the characteristic time representing the changes in MSD, respectively. Furthermore, the spectrum *h*(τ) can be written as
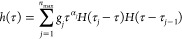
14where *n*_max_ is the number of *j* modes, with each mode having a relaxation time τ_*j*_ (τ_*j*_ > τ_*j*–1_) and exponent α_*j*_. *H*(τ) is the Heaviside step function. The function in the first term is
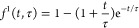
15

The function in the second term is
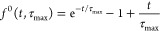
16which is weighted by the constant *g*_0_ = ∑_*j* = 1_^*n*_max_^*g*_*j*_(τ_*j*_^α_*j*_^ – τ_*j* – 1_^α_*j*_^)/α_*j*_, ensuring that the first term in eq 13 equals the second term when *t* = τ_max_. The other weighted terms include  for 2 ≤ *j* ≤ *n*_max_. The value of *g*_1_ can be obtained from 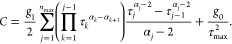 Given that τ_max_ is usually large, the second term in the expression for *C* is negligible, and once the slope of the ballistic regime is known, *g*_1_ and other weighted terms can be readily computed. The integration of eqs 13 with respect to τ then gives rise to
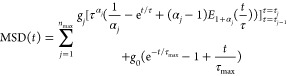
17where  is the exponential integral function. The Fourier transform of eq 17 gives the real and imaginary parts of the MSD in the frequency domain as
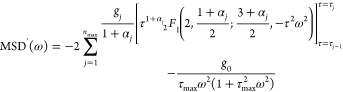
18
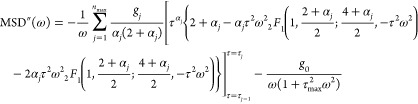
19where _2_*F*_1_ is the hypergeometric function and [*x*(τ)]_τ=τ_*j*–1__^τ=*τ*_*j*_^ = *x*(τ_*j*–1_) – *x*(τ_*j*_) for an arbitrary function *x*(τ). The parameters {α_1_, ···, α_max_,τ_0_, τ_1_, ···, τ_max_} are obtained based on the fit to the probe’s MSD data. We fixed *τ*_*j*_ values and numerically sought α_*j*_ values that minimize χ^2^ of the MSD, which were then used to obtain the MSD values in the frequency domain (eqs 18 and 19).

## Supporting Information Available

Amino acid codes of E–K and A1-LCD sequence variants, dense phase concentration, radial distribution function between probe and protein chains, mean square displacement of the probe in condensates of proteins with different chain lengths, viscoelastic modulus obtained based on single vs multiple probes, comparison of viscoelastic modulus obtained from different rheology techniques, probe’s radius of gyration and relative anisotropy, shear stress relaxation modulus, potential energy curves, and a simulation snapshot highlighting probe–protein interactions (PDF)

## Notes

Analysis codes, input scripts, and initial and final configuration files for conducting passive rheology simulations of single-component and multicomponent condensates are available via the GitHub repository: https://github.com/dsd993/PassiveRheologyCondensates-Sims-Analysis.
