# Supplementary material for: Sequence-Encoded Spatiotemporal Dependence of Viscoelasticity of Protein Condensates Using Computational Microrheology
Source: JACS Au. 2024 Nov 11;4(11):4394–405. doi: 10.1021/jacsau.4c00740 (PMC11600178; doi:10.1021/jacsau.4c00740)
Supplement: Supplementary file 1 — au4c00740_si_001.pdf [file au4c00740_si_001.pdf]

Supplementary Information

Sequence-encoded Spatiotemporal Dependence of Viscoelasticity of Protein Condensates Using Computational Microrheology

Dinesh Sundaravadivelu Devarajan,[1,*] and Jeetain Mittal[1,2,3,*]

[1]Artie McFerrin Department of Chemical Engineering, Texas A&M University, College Station, TX 77843, United States

[2]Department of Chemistry, Texas A&M University, College Station, TX 77843, United States

[3]Interdisciplinary Graduate Program in Genetics and Genomics, Texas A&M University, College Station, TX 77843, United States

[*]Corresponding authors' email: dineshsd@tamu.edu, jeetain@tamu.edu

Table S1. Amino acid codes of E−K sequence variants of chain length $N = 250$ that were used in this study.

E−K	Sequence
nSCD = 0.067	EEKEEEEKEKEEEEKEEKKEKKKEEKEKEEKKEKEKEKEKEEEEEKKEEEEEEEEE<br>KEKEEEEKKKKKKEEEEEKKKEEKKKEKKEEEKEEEKEKKEEKEEEEEEEEKEEEE<br>EKKEEKEKEEKKKEKEEKEKEEKKEKKKEKKKKEEEKKKEKEKKKEKKKEEKEEKE<br>KEKKKKEKEKEKKEKEKKEKEKKKKEKKKKEEKEKKEEEKKKEKEKKKKKKEKEKK<br>KKKEEKKKKKKEEEKKEEKKKEKKEE
nSCD = 0.468	EEEEEEEEEEEEEEKEEEEEEEEEEEEKEEEEEEEEEEEEEKEEEEEEEEKEEEEEE<br>EKEEEEEEKEEKKKEEKEKEKEEEEKEKEEEEEKEEEEKKEEEEEEEEKEEEEEEE<br>EEEEEKKEKKEKKKEEKEKKEKKEKKKKKEKKKKEKKKKKKEKKKKKKEKEKEKEK<br>KEKKKKKKKKKEEKKKKEKKKKKKKKKKKEEKKKKKKEKKKKKKKKKEKKKKKKKK<br>KKKKKKKEKEKKKKKKKKEEKKKKKK
nSCD = 1.000	EEEEEEEEEEEEEEEEEEEEEEEEEEEEEEEEEEEEEEEEEEEEEEEEEEEEEEEE<br>EEEEEEEEEEEEEEEEEEEEEEEEEEEEEEEEEEEEEEEEEEEEEEEEEEEEEEEE<br>EEEEEEEEEEEEEKKKKKKKKKKKKKKKKKKKKKKKKKKKKKKKKKKKKKKKKKKK<br>KKKKKKKKKKKKKKKKKKKKKKKKKKKKKKKKKKKKKKKKKKKKKKKKKKKKKKKK<br>KKKKKKKKKKKKKKKKKKKKKKKKKK

Table S2. Amino acid codes of A1-LCD wildtype (WT) and its sequence variants of chain length $N = 137$ that were used in this study.

A1-LCD	Sequence
**WT**	GSMASASSSQRGRSGSGNFGGGRGGGFGGNDNFGRGGNFSGRGGFGGSRGGGGYGG SGDGYNGFGNDGSNFGGGGSYNDFGNYNNQSSNFGPMKGGNFGGRSSGPYGGGGQY FAKPRNQGGYGGSSSSSSYGSGRRF
**allY**	GSMASASSSQRGRSGSGNYGGGRGGGYGGNDNYGRGGNYSGRGGYGGSRGGGGYGG SGDGYNGYGNDGSNYGGGGSYNDYGNYNNQSSNYGPMKGGNYGGRSSGPYGGGGQY YAKPRNQGGYGGSSSSSSYGSGRRY
**allW**	GSMASASSSQRGRSGSGNWGGGRGGGWGGNDNWGRGGNWSGRGGWGGSRGGGGWGG SGDGWNGWGNDGSNWGGGGSWNDWGNWNNQSSNWGPMKGGNWGGRSSGPWGGGGQW WAKPRNQGGWGGSSSSSSWGSGRRW
**allF**	GSMASASSSQRGRSGSGNFGGGRGGGFGGNDNFGRGGNFSGRGGFGGSRGGGGFGG SGDGFNGFGNDGSNFGGGGSFNDFGNFNNQSSNFGPMKGGNFGGRSSGPFGGGGQF FAKPRNQGGFGGSSSSSSFGSGRRF

Table S3. Viscosity η of the dense phase of the condensates formed by A1-LCD wildtype (WT) and its sequence variants from computational microrheology and experimental microrheology methods. In both simulations and experiments, η increased in the following order: allW $>$ allY $\approx$ WT $>$ allF.

A1-LCD	η (mPa.s) from microrheology simulations (this work, $T = 245K$)	η (Pa.s) from microrheology experiments (room temperature)
**WT**	1.040 ± 0.008	1.247 ± 0.804
**allY**	1.121 ± 0.009	1.736 ± 1.237
**allW**	1.756 ± 0.017	12.143 ± 6.231
**allF**	0.748 ± 0.003	0.227 ± 0.141

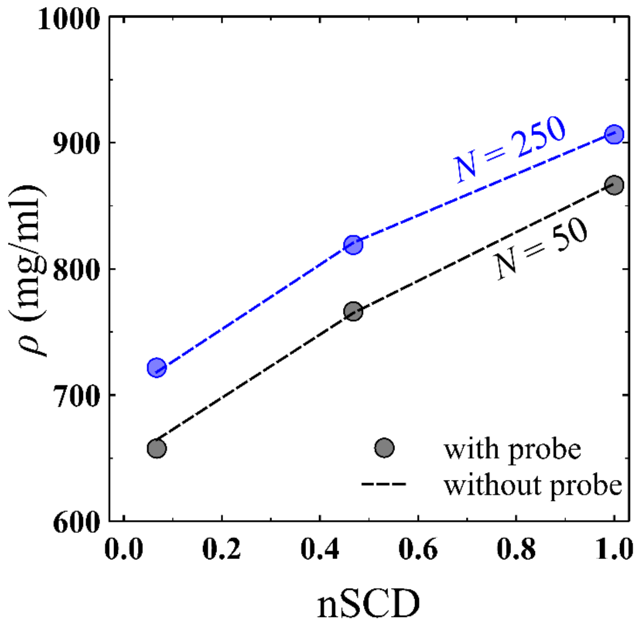

Fig. S1. Dense phase concentration ρ attained by the E−K sequence variants of chain lengths $N = 50$ and $N = 250$ with and without the presence of a probe particle in the system.

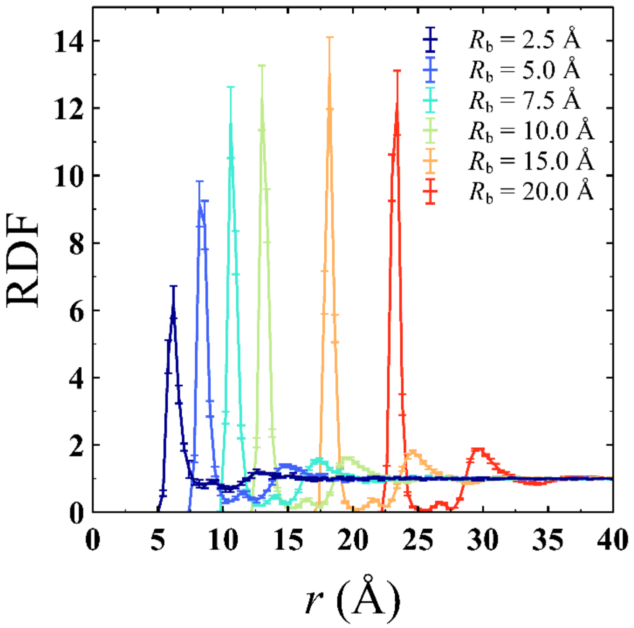

Fig. S2. Radial distribution function RDF between the center of mass of the probe and the residues of the protein chains as a function of probe's bare radius R_b for the E−K sequence variants of chain length $N = 250$.

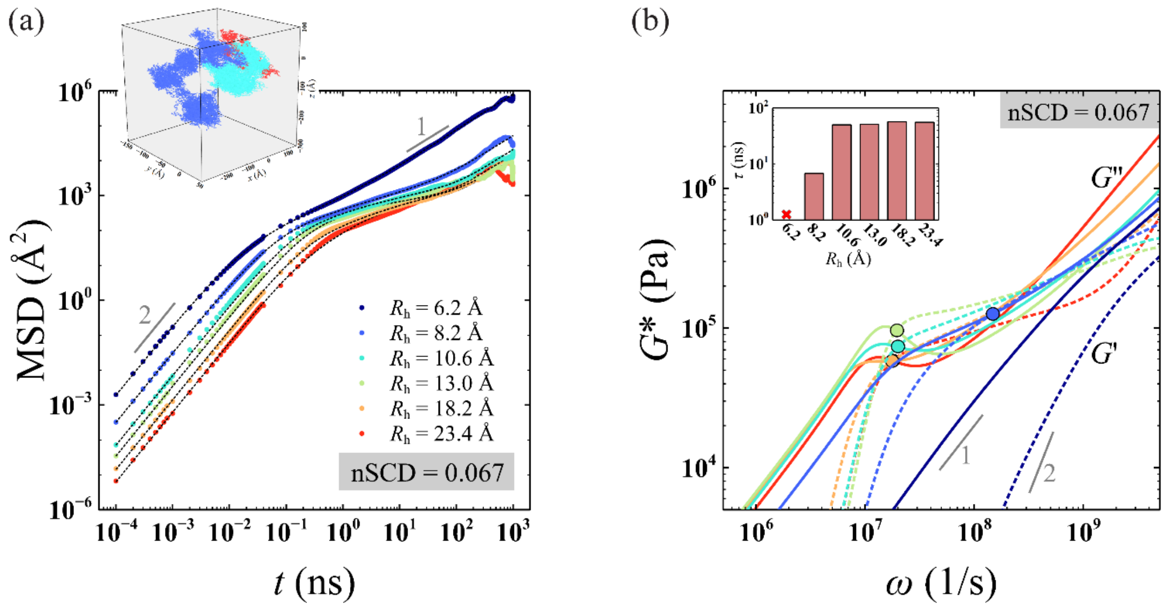

Fig. S3. (a) Mean square displacement $\text{MSD}(t)$ of the probe particle of different hydrodynamic radius R_{h} in the dense phase of the E−K sequence with nSCD = 0.067. The dashed lines are the fits based on the Baumgaertel-Schausberger-Winter-like power law spectrum to the MSD data. The inset shows probe particle's displacement in the three-dimensional Cartesian coordinates for select R_{h} values. (b) Elastic G' (dashed line) and viscous G'' (solid line) modulus for the dense phase of the E−K sequence with nSCD = 0.067 as a function of R_{h}, with circles corresponding to the crossover frequency. The inset shows the relaxation time τ with varying R_{h}.

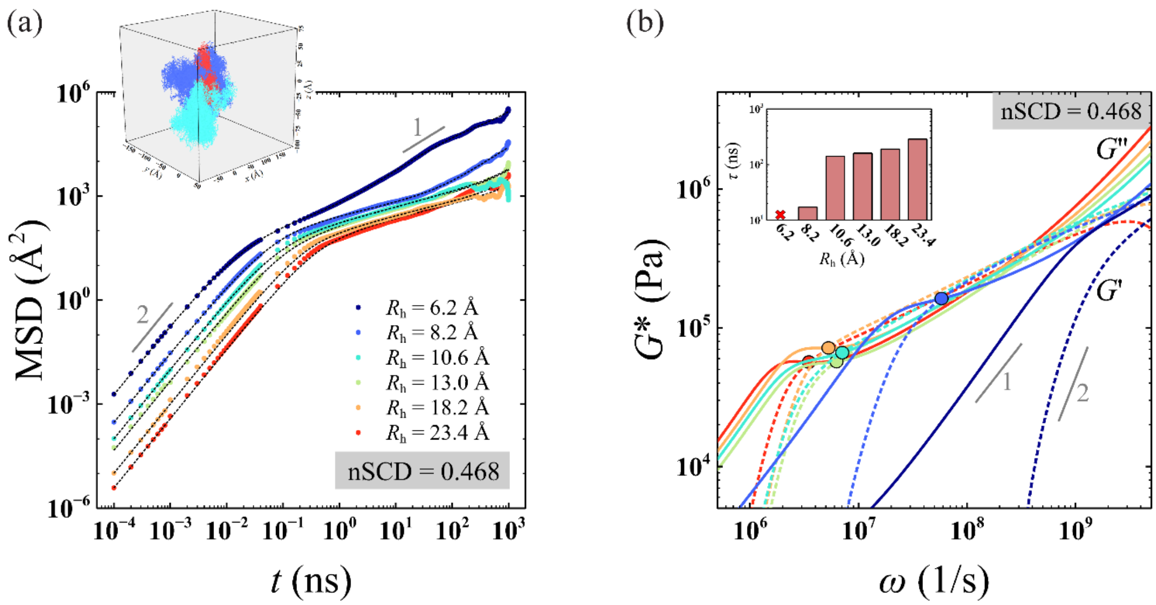

Fig. S4. (a) Mean square displacement $\mathrm{MSD}(t)$ of the probe particle of different hydrodynamic radius R_h in the dense phase of the E−K sequence with nSCD = 0.468. The dashed lines are the fits based on the Baumgaertel-Schausberger-Winter-like power law spectrum to the MSD data. The inset shows probe particle's displacement in the three-dimensional Cartesian coordinates for select R_h values. (b) Elastic G' (dashed line) and viscous G'' (solid line) modulus for the dense phase of the E−K sequence with nSCD = 0.468 as a function of R_h, with circles corresponding to the crossover frequency. The inset shows the relaxation time τ with varying R_h.

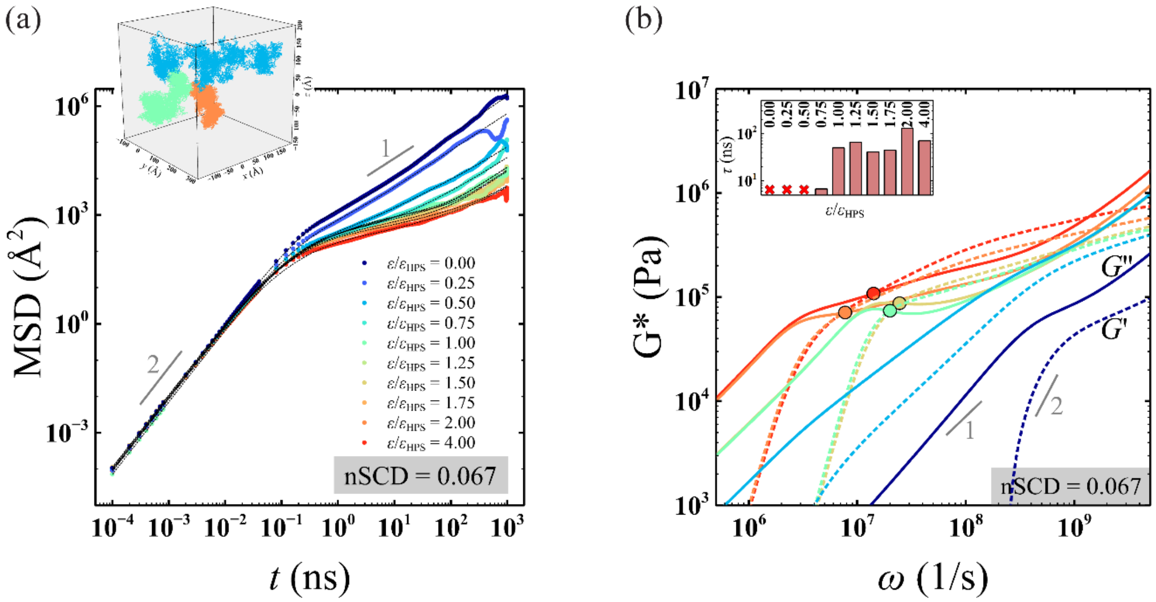

Fig. S5. (a) Mean square displacement MSD(t) of the probe particle for different normalized probe-protein interaction strength $\varepsilon/\varepsilon_{\text{HPS}}$, where $\varepsilon_{\text{HPS}} = 0.20$ kcal/mol, in the dense phase of the E-K sequence with nSCD $= 0.067$. The dashed lines are the fits based on Baumgaertel-Schausberger-Winter-like power law spectrum to the MSD data. The inset shows probe particle's displacement in the three-dimensional Cartesian coordinates for select $\varepsilon/\varepsilon_{\text{HPS}}$ values. (c) Elastic G' (dashed line) and viscous G'' (solid line) modulus for the dense phase of the E−K sequence with nSCD $= 0.067$ as a function of $\varepsilon/\varepsilon_{\text{HPS}}$, with circles corresponding to the crossover frequency. The inset shows the relaxation time τ with varying $\varepsilon/\varepsilon_{\text{HPS}}$.

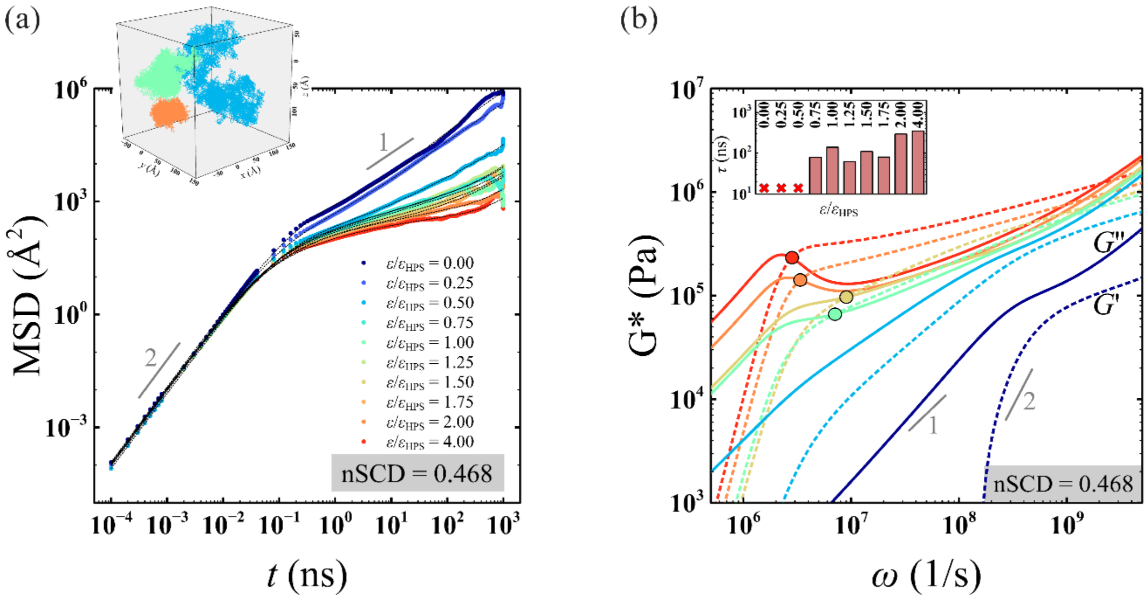

Fig. S6. (a) Mean square displacement MSD(t) of the probe particle for different normalized probe-protein interaction strength $\varepsilon/\varepsilon_{\mathrm{HPS}}$, where $\varepsilon_{\mathrm{HPS}} = 0.20$ kcal/mol, in the dense phase of the E-K sequence with nSCD = 0.468. The dashed lines are the fits based on Baumgaertel-Schausberger-Winter-like power law spectrum to the MSD data. The inset shows probe particle's displacement in the three-dimensional Cartesian coordinates for select $\varepsilon/\varepsilon_{\mathrm{HPS}}$ values. (c) Elastic G' (dashed line) and viscous G'' (solid line) modulus for the dense phase of the E−K sequence with nSCD = 0.468 as a function of $\varepsilon/\varepsilon_{\mathrm{HPS}}$, with circles corresponding to the crossover frequency. The inset shows the relaxation time τ with varying $\varepsilon/\varepsilon_{\mathrm{HPS}}$.

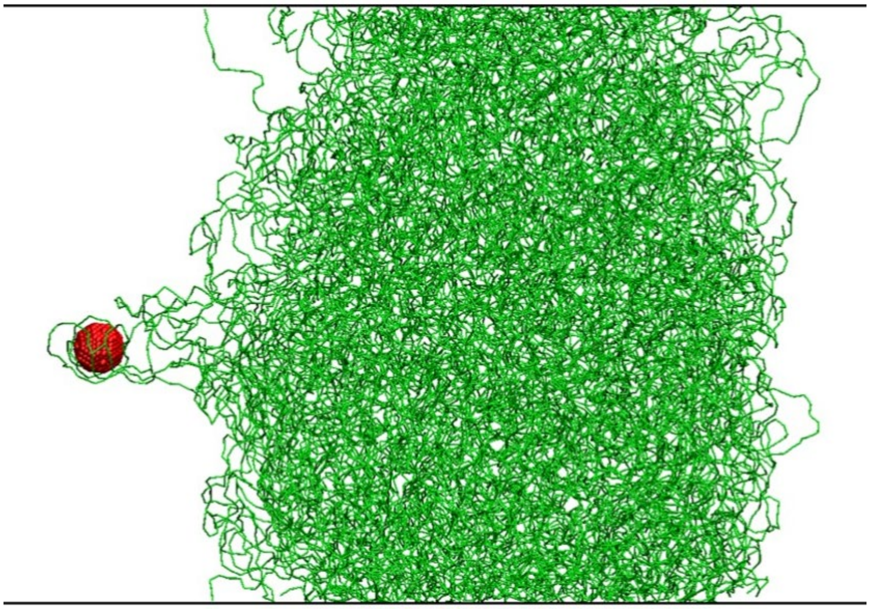

Fig. S7. Simulation snapshot showing strong adsorption of E−K protein chains (green) to the probe particle (red) surface, when $\varepsilon/\varepsilon_{\mathrm{HPS}} = 2$, where $\varepsilon_{\mathrm{HPS}} = 0.20$ kcal/mol.

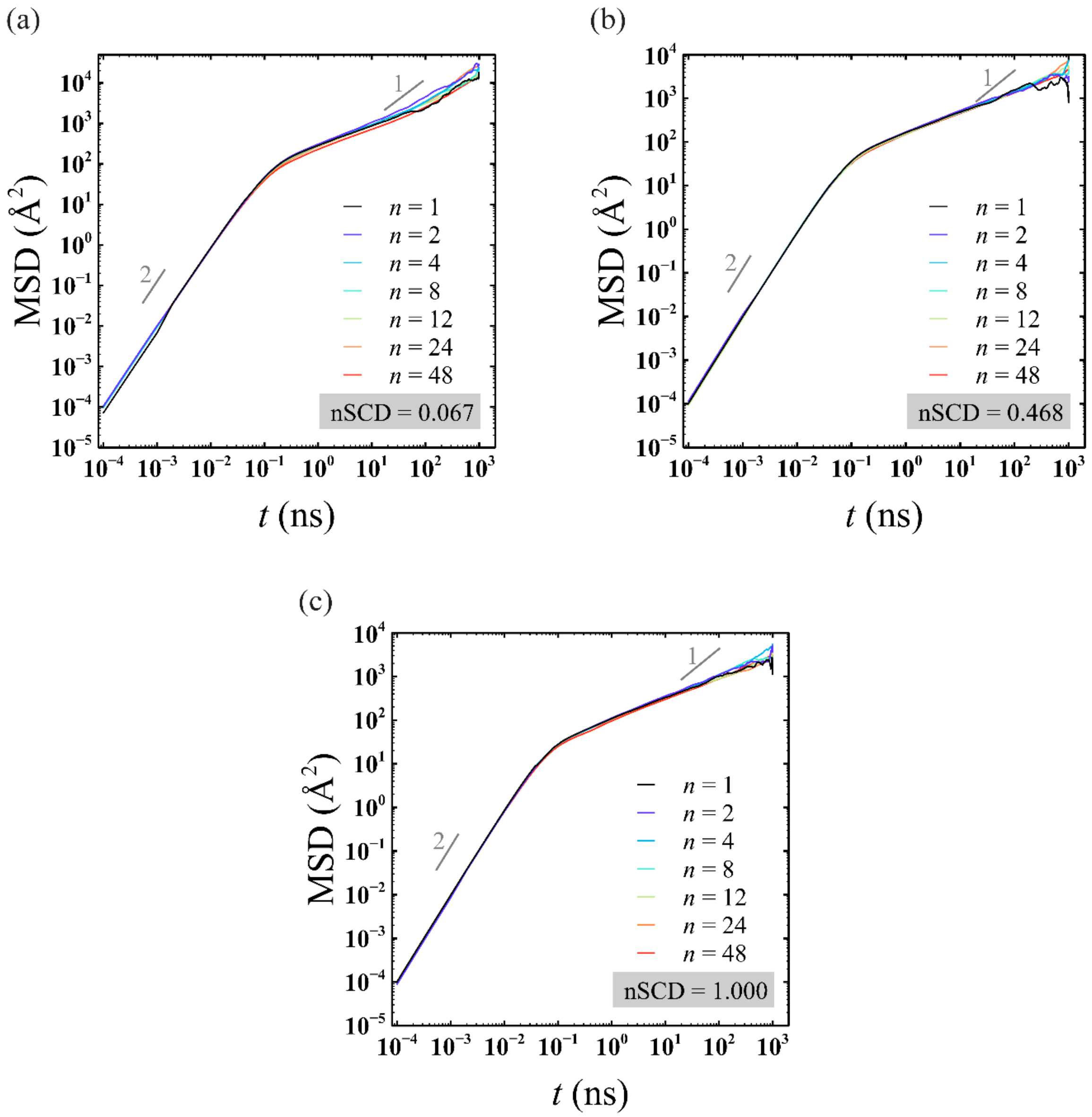

Fig. S8. (a) Average mean square displacement MSD(t) of the probe varying in number n in the dense phase of the E−K sequences for (a) nSCD = 0.067, (b) nSCD = 0.468, and (c) nSCD = 1.000, respectively.

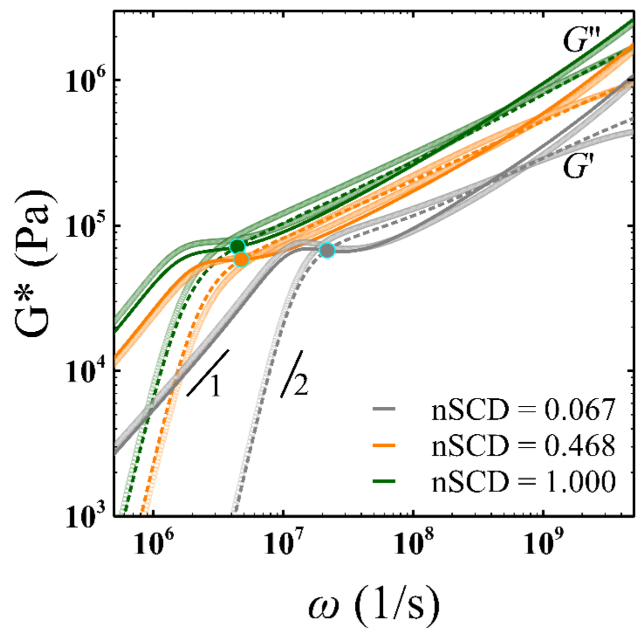

Fig. S9. Elastic G' and viscous G'' modulus for the E−K sequences with different nSCD. The lines correspond to those obtained based on multiple ($n = 8$) probes and the symbols correspond to those obtained based on a single probe in each dense phase system.

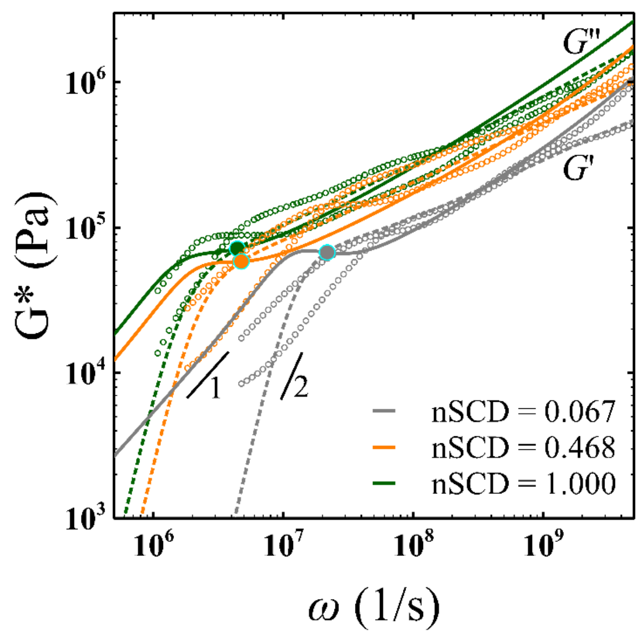

Fig. S10. Elastic G' and viscous G'' modulus for the E−K sequences with different nSCD. The lines correspond to those obtained based on multiple ($n = 8$) probes from microrheology simulations and the symbols correspond to those obtained from the Fourier transform of shear stress relaxation modulus $G(t)$ computed using the Green-Kubo (GK) relation for each dense phase system.

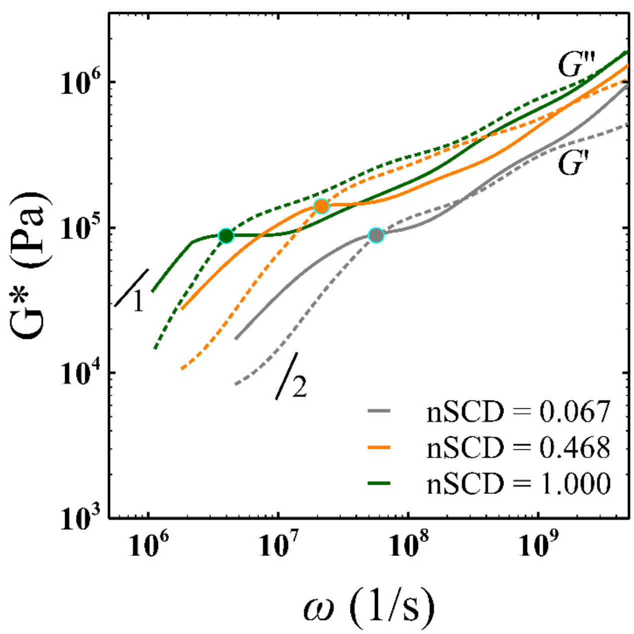

Fig. S11. Elastic G' (dashed line) and viscous G'' (solid line) modulus for the dense phase of the E−K sequences with different nSCD, obtained from the Fourier transform of shear stress relaxation modulus $G(t)$ computed using the Green-Kubo (GK) relation.

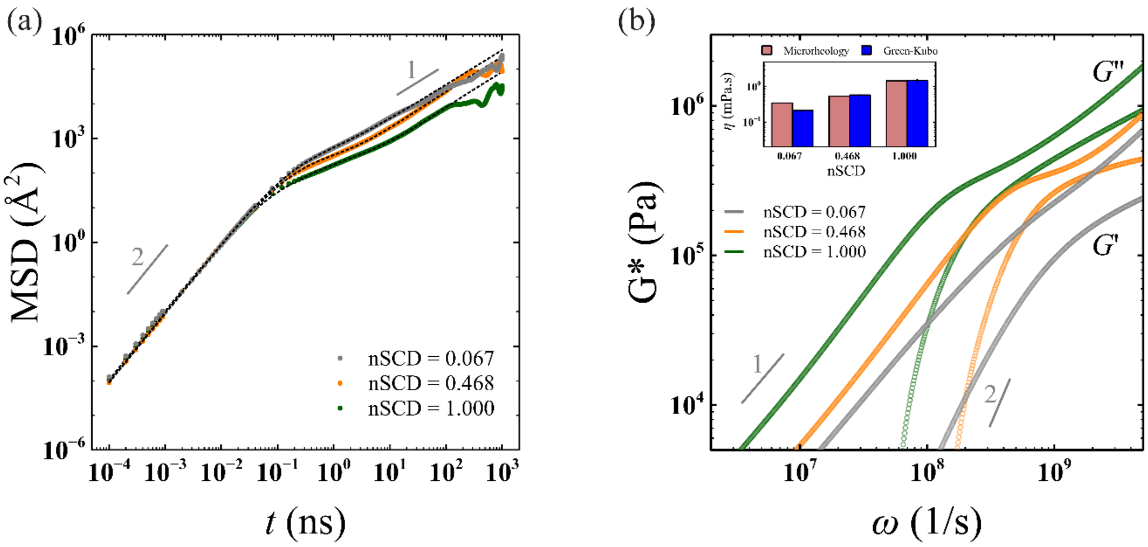

Fig. S12. (a) Mean square displacement MSD(t) of the probe particle in the dense phase of different nSCD sequences of $N = 50$. The dashed lines are the fits based on Baumgaertel-Schausberger-Winter-like power law spectrum to the MSD data. (b) Elastic G' and viscous G'' modulus for the dense phase of different nSCD sequences of $N = 50$. The insets shows the comparison between the viscosity η values obtained from the microrheology simulations and the Green-Kubo method.

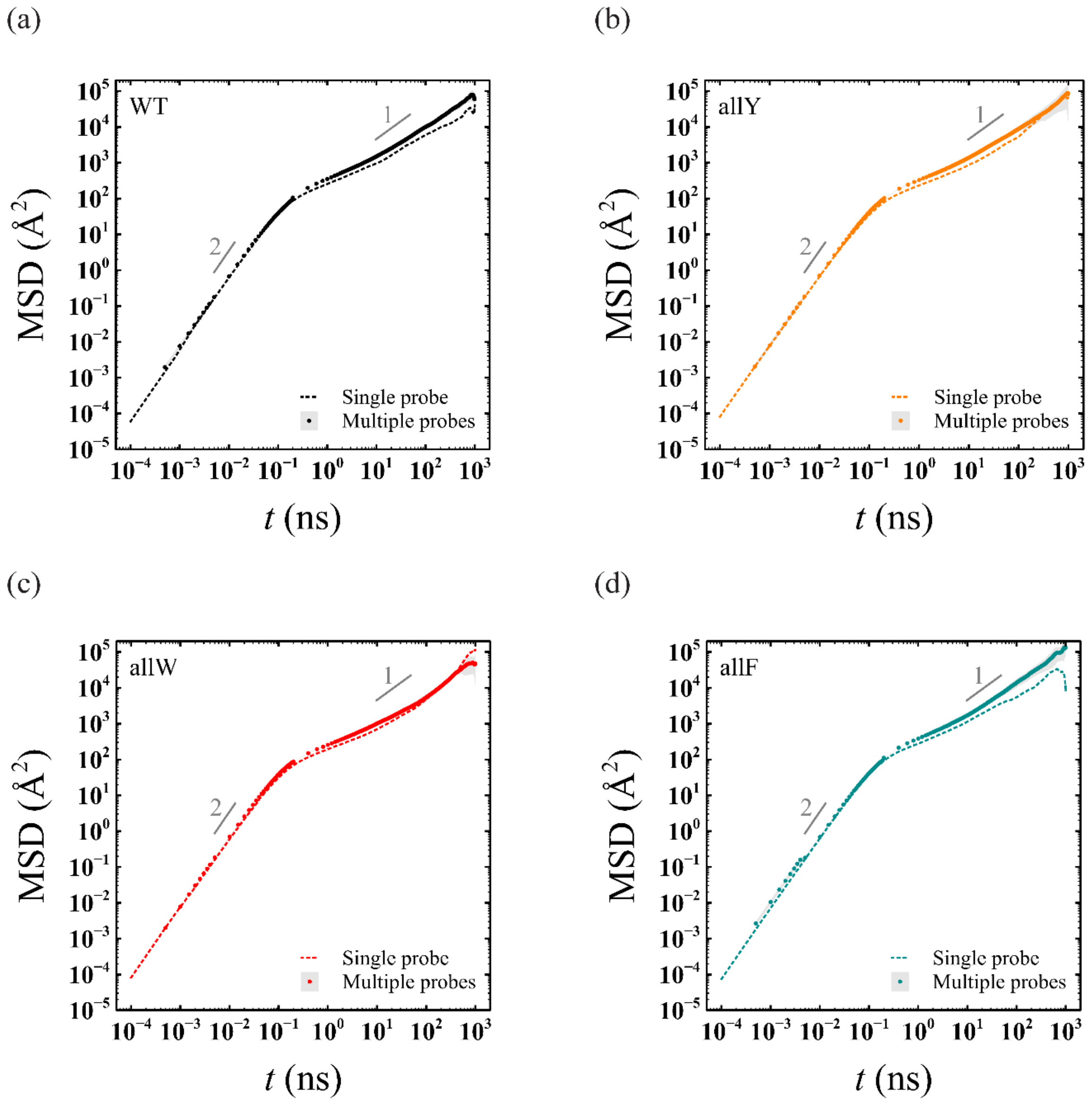

Fig. S13. (a) Mean square displacement $\mathrm{MSD}(t)$ of a single probe particle compared with that obtained as an average based on multiple ($n = 6$) probes in the dense phase of the (a) A1-LCD WT sequence and its variants (b) allY, (c) allW, and (d) allF, respectively.

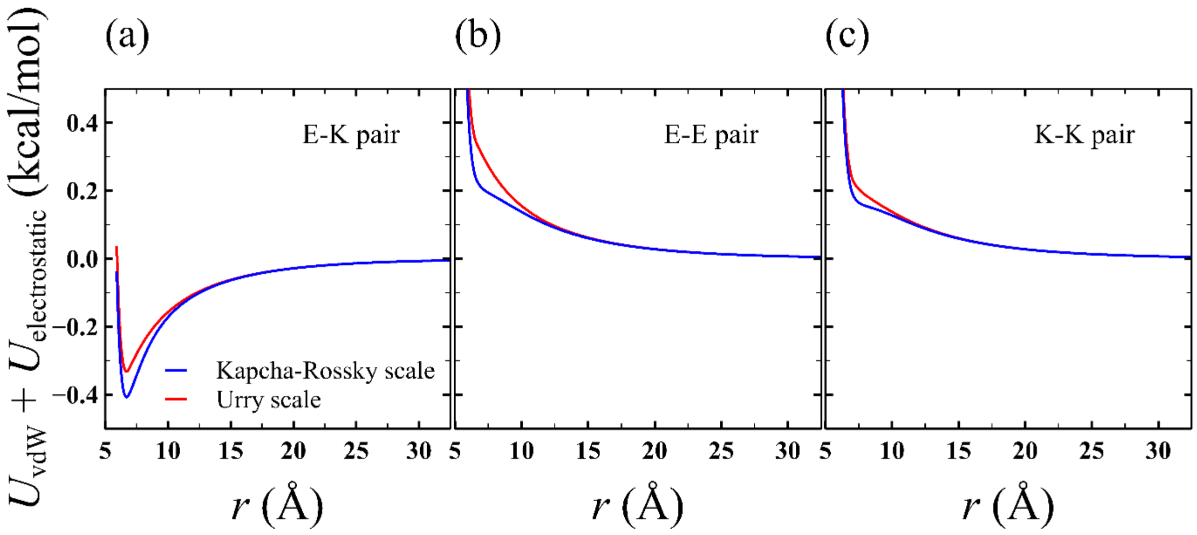

Fig. S14. Potential energy curves (van der Waals vdW + electrostatic interactions) for (a) oppositely charged residues and (b, c) like charged residues in E−K sequences.

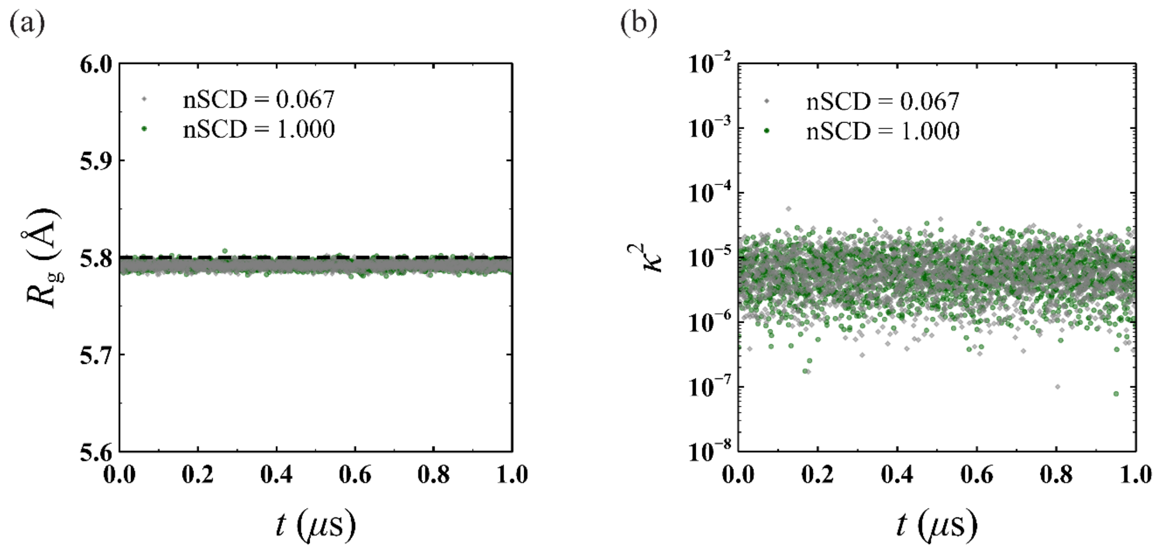

Fig. S15. Time evolution of (a) radius of gyration R_g and (b) relative anisotropy κ^2 for the probe particle of bare radius $R_\mathrm{b} = 7.5$ Å undergoing Brownian motion in the dense phase of E−K sequences with nSCD = 0.067 and nSCD = 1.000, respectively. The dashed line in (a) corresponds to the expected values for a spherical particle $R_\mathrm{g} = \sqrt{3R_\mathrm{b}^2/5}$.

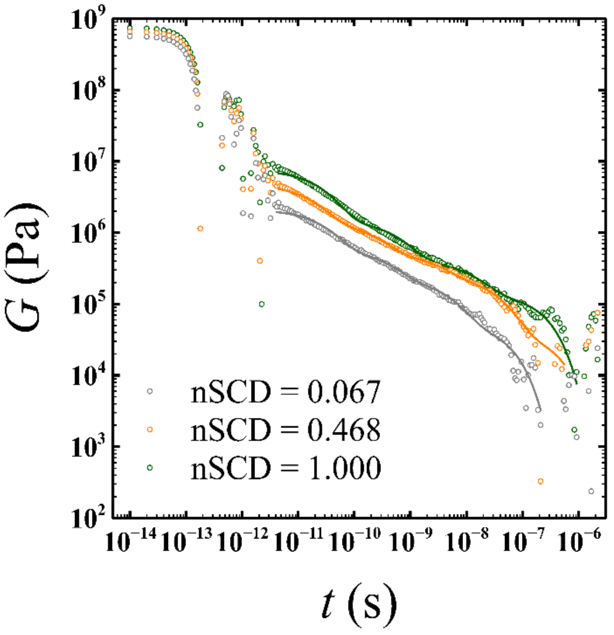

Fig. S16. Shear stress relaxation modulus $G(t)$ obtained using the Green-Kubo (GK) relation for the E−K sequence variants with different nSCD. The solid lines at long time scales represent the fit to a series of Maxwell modes.
